# Praktikable Sjögren-Diagnostik bei interstitieller Lungenerkrankung – ein Diskussionsbeitrag

**DOI:** 10.1007/s00393-023-01344-3

**Published:** 2023-03-22

**Authors:** Martin Aringer, Dirk Koschel, Thomas Dörner, Philipp Sewerin, Antje Prasse, Torsten Witte

**Affiliations:** 1 Bereich Rheumatologie, Medizinische Klinik und Poliklinik III, Universitätsklinikum und Medizinische Fakultät Carl Gustav Carus, TU Dresden, Fetscherstr. 74, 01307 Dresden, Deutschland; 2 UniversitätsCentrum für Autoimmun- und Rheumatische Erkrankungen (UCARE), Universitätsklinikum und Medizinische Fakultät Carl Gustav Carus, TU Dresden, Dresden, Deutschland; 3 Bereich Pneumologie, Medizinische Klinik und Poliklinik I, Universitätsklinikum und Medizinische Fakultät Carl Gustav Carus, TU Dresden, Dresden, Deutschland; 4 Medizinische Klinik mit Schwerpunkt Rheumatologie und Klinische Immunologie, Charité Universitätsmedizin Berlin, Berlin, Deutschland; 5 Rheumazentrum Ruhrgebiet, Herne, Deutschland; 6 Klinik für Pneumologie, Medizinische Hochschule Hannover, Hannover, Deutschland; 7 Klinik für Rheumatologie und Immunologie, Medizinische Hochschule Hannover, Hannover, Deutschland; 8 Abteilung für Innere Medizin/Pneumologie, Fachkrankenhaus Coswig, Coswig, Deutschland; 9 Ruhr-Universität Bochum, Bochum, Deutschland

**Keywords:** Sjögren-Syndrom, Interstitielle Lungenerkrankung, Sicca-Symptomatik, Antinukleäre Antikörper, Speicheldrüsensonographie, Sjögren’s syndrome, Interstitial lung disease, Sicca symptoms, Antinuclear antibodies, Salivary gland sonography

## Abstract

Das Sjögren-Syndrom (SjS) stellt eine mögliche autoimmune Ursache einer interstitiellen Lungenerkrankung dar. Die Abklärung in Richtung SjS ist aber im Vergleich zu anderen systemischen Autoimmunerkrankungen bisher kaum standardisiert. Die subjektive Sicca-Symptomatik, die Anti-SS-A/Ro-Antikörper und selbst die ANA-Diagnostik als Suchtest haben alle relevante Einschränkungen in ihrer Sensitivität und/oder Spezifität. Vor diesem Hintergrund haben wir in einer interdisziplinären Diskussion einen Konsens für die SjS-Abklärung entwickelt, den wir hier für die breitere Diskussion vorstellen. Neben ANA sollten sowohl Anti-SS-A/Ro-Antikörper als auch Antikörper gegen α‑Fodrin bestimmt werden. Wichtig ist die Objektivierung der Trockenheit mittels Schirmer- und Saxon-Test und bei fehlenden typischen Autoantikörpern die Speicheldrüsenbiopsie.

## Abstract (en)

Sjögren’s syndrome (SjS) is a possible autoimmune cause of interstitial lung disease. The diagnostic pathway for SjS, however, is largely undefined in comparison to other systemic autoimmune diseases. Subjective sicca symptoms, anti-SS-A/Ro antibodies and even ANA as screening tests all have relevant limitations in sensitivity and/or specificity. Against this background, in an interdisciplinary discussion we have developed a consensus for the clarification of SjS, which is presented here for broader discussion. In addition to ANA and anti-SS-A/Ro antibodies, antibodies against alpha-fodrin should be included. Objective measures of dryness, such a Schirmer and Saxon tests are important, as is a salivary gland biopsy in the absence of typical autoantibodies.

Neben der systemischen Sklerose (SSc) und Mischkollagenose (MCTD), Anti-Synthetasesyndromen und bestimmten Dermatomyositiden, der rheumatoiden Arthritis (RA) sowie der mikroskopischen Polyangiitis (MPA) kommt auch das primäre Sjögren-Syndrom (SjS) als Ursache einer interstitiellen Lungenerkrankung (ILD) infrage [1]. Während die ILD-Endstrecke der Fibrosierung für die meisten Erkrankungen ähnlich ist und für deren Beeinflussung bei progredient-fibrotischem Verlauf nur eine zugelassene Therapie, nämlich Nintedanib [2], zur Verfügung steht, hängt der gezielte Einsatz immunsuppressiver Medikamente oder immunmodulierender Basistherapien [3, 4] von der richtigen Diagnose ab.

Für die meisten der systemischen Autoimmunerkrankungen sind die diagnostischen Wege gut definiert. Zur Abklärung einer unklaren ILD gehört heute neben ANA in der indirekten Immunfluoreszenz auf HEp-2-Zellen, pANCA/Anti-MPO-Antikörpern (für die MPA) und Anti-CCP-Antikörpern (für die seropositive RA) der Myositisblot. SSc und MCTD verursachen neben meist positiven ANA die typische, schwere Raynaud-Symptomatik.

In Richtung SjS ist die Abklärung hingegen deutlich weniger gut standardisiert und vereinheitlicht [5]. Prävalenzschätzungen für das SjS zeigen eine große Bandbreite von etwa 1:1000 bis etwa 1:100 [6]. Anamnestische Angaben zur Sicca-Symptomatik sind leider unverlässlich, und eine klinisch eindeutige Schwellung von Speicheldrüsen ist eher die Ausnahme als die Regel. Dazu kommen Unsicherheiten bezüglich der Autoantikörper. Die Anti-Ro-Antikörper sind ein wesentlicher Befund für die Klassifikation des SLE [7]. Bei Weitem nicht alle SjS-Patientinnen und Patienten haben positive Anti-SS-A/Ro-Antikörper, und ein Teil der anti-SS-A/Ro-positiven Patientinnen und Patienten hat dennoch negative ANA und nur ein zytoplasmatisches Fluoreszenzsignal, das mitunter nicht berichtet wird.

Vor diesem Hintergrund kamen die Autoren auf Einladung der Firma Boehringer-Ingelheim zu einer virtuellen Diskussionsrunde zusammen und versuchten, gemeinsam einen umsetzbaren Standard einer Abklärung in Richtung SjS bei unklarer ILD zu entwickeln. Die Firma stellte zwar die Plattform zur Verfügung, nahm aber keinerlei Einfluss auf die Diskussion und ist am vorliegenden Manuskript in keiner Weise beteiligt. Da am Schluss der Sitzung ein Konsens über sinnvolle Maßnahmen erzielt werden konnte, halten wir diese Ergebnisse für einen sinnvollen Diskussionsbeitrag.

Die Diskussion wurde von Vorträgen aus pneumologischer Sicht (DK) und rheumatologischer Sicht (TW) eingeleitet, um einen gemeinsamen Faktenstand zu gewährleisten. Pneumologisch kann sich das Sjögren-Syndrom unterschiedlich äußern. Neben den relativ SjS-typischen Befunden der lymphozytischen interstitiellen Pneumonie (LIP) und Zysten kommen sowohl eine ILD mit einem Muster einer nichtspezifischen interstitiellen Pneumonie (NSIP) als auch ein UIP („usual interstitial pneumonia“)-Muster infrage sowie auch eine organisierende Pneumonie (OP-Muster) [8]. Nicht selten bestehen auch Mischbilder aus den genannten Mustern.

Aus rheumatologischer Sicht wurde auf die Möglichkeit des ANA-negativen SjS hingewiesen, das sowohl bei Anti-SS-A/Ro-Antikörpern als falsch negativer Befund als auch bei Patientinnen und Patienten mit Anti-α-Fodrin-Antikörpern [9, 10] möglich ist. So hatten in der Hannoveraner SjS-ILD-Kohorte von 31 Patientinnen und Patienten 3 keine höhertitrigen ANA (> 1:160), 10 (32 %) keine Anti-SS-A-Antikörper und 24 (77 %) keine Anti-SS-B-Antikörper [8]. Ebenso wurden die Unverlässlichkeit anamnestischer Angaben zur Sicca-Symptomatik und die Notwendigkeit der Objektivierung durch Schirmer- und Saxon-Test sowie Speicheldrüsensonographie (Tab. 1) dokumentiert [6].

**Table 1:** 

	Bemerkungen
*Immunologie*	
ANA	Immunfluoreszenz auf HEp-2-Zellen
Anti-SS-A/Ro-Antikörper	Sowohl Antikörper gegen Ro-60 (SS-A) als auch Ro-52
Anti-α-Fodrin-Antikörper	–
*Screeningfragen*	
Rote Augen	Alternativ täglich befeuchtende Augentropfen
Zahn- oder Zahnfleischprobleme	–
*Objektivierung der Trockenheit*	
Schirmer-Test	Filterpapierstreifen konjunktival ≤ 5 mm feucht in 5 min
Saxon-Test	Gewichtszunahme Tupfer beim Kauen ≤ 2,75 g in 2 min
*Objektivierung der Sialadenitis*	
Biopsie kleiner Speicheldrüsen	Lymphozytäre Infiltrate; mindestens 1 Fokus/4 mm^2^
Sonographie der Speicheldrüsen	–

In Anbetracht der serologischen Unsicherheiten empfehlen wir zusätzlich zu den ANA auch die Bestimmung der Anti-SS-A/Ro- und der Anti-α-Fodrin-Antikörper (Abb. 1). IgA-Antikörper gegen α‑Fodrin finden sich bei fast zwei Drittel (64 %) der Sera von Patientinnen und Patienten mit SjS, aber in weniger als 1 % aller gesunden Spendersera [11]. Der Test ist kommerziell verfügbar (Aesku Diagnostics, Wendelsheim, Germany, Orgentec Diagnostics, Mainz, Germany).

**Figure 1:**
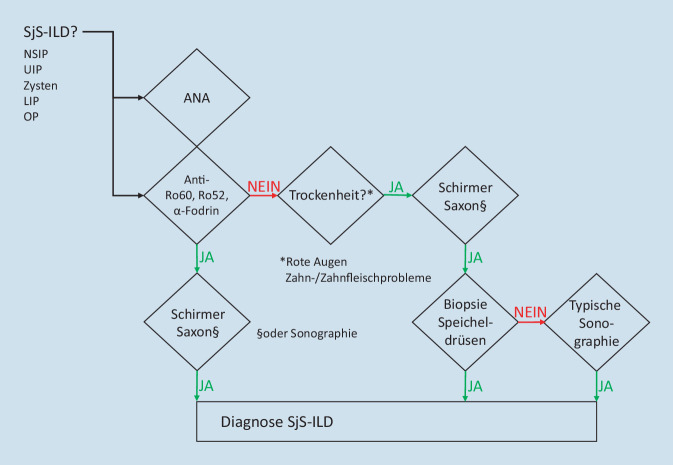

Sind Anti-SS-A/Ro-Antikörper, Anti-α-Fodrin-Antikörper oder hochtitrig positive ANA vorhanden, sollte mittels Schirmer-Test (und/oder Bengalrosa) eine okuläre Sicca-Symptomatik und mittels Saxon-Test [12] und/oder Speicheldrüsensonographie [13, 14] eine orale Sicca-Symptomatik bzw. Speicheldrüsenentzündung gesucht werden. Bei positiven Anti-SS-A/Ro-Antikörpern kommen differenzialdiagnostisch auch der systemische Lupus erythematodes (SLE) und seltener SSc oder Myositiden infrage.

Sind die Antikörper negativ, empfehlen wir zusätzlich, nach regelmäßig roten Augen und nach Zahn- und Zahnfleischproblemen zu fragen (Abb. 1), weil reines Trockenheitsgefühl als Screeningfrage weder sensitiv noch spezifisch ist. Positive Antworten sollten auch hier zur Objektivierung (des jeweiligen Symptoms) führen. Eine objektivierte Sicca-Symptomatik ohne Antikörper gegen SS-A/Ro oder α‑Fodrin sollte mittels Speicheldrüsenbiopsie weiter abgeklärt werden (Abb. 1). Auch bei negativer Biopsie spricht eine typische Sonographie für ein SjS, ist aber aus heutiger Sicht nicht so spezifisch wie die Biopsie.

Grundsätzlich kommen diese differenzialdiagnostischen Überlegungen auch bei anderen Situationen zum Tragen. Während bei der ILD und vielleicht auch bei der interstitiellen Nephritis das Sjögren-Syndrom nur eine von unterschiedlichen möglichen Ursachen darstellt, wird dann jedoch die Ausgangslage das SjS meist sehr viel eindeutiger nahelegen. Das Schema in Abb. 1 ist aber bewusst so gestaltet, dass auch ein Einstieg an anderen Positionen möglich ist.

Zusammenfassend haben wir in einer relativ kleinen Expertengruppe einen Konsensus für die SjS-Abklärung erzielt, den wir für ausreichend sensitiv und praktikabel halten. Auch wenn sowohl die Pneumologie als auch die Rheumatologie inhaltlich sehr gut vertreten waren, kann das in Anbetracht der begrenzten Zahl an Teilnehmerinnen und Teilnehmern und der nur bedingt systematischen Literaturberücksichtigung nur als Vorschlag für die breitere Diskussion dienen. Wir hoffen aber, dass die kurze Darstellung in dieser Funktion nützlich ist.
